# From Symptomatology to Functioning - Applying the ICF to Autism Measures to Facilitate Neurodiversity-Affirmative Data Harmonization

**DOI:** 10.1007/s10803-023-06204-2

**Published:** 2023-12-11

**Authors:** Melissa H Black, Karl Lundin Remnélius, Lovisa Alehagen, Thomas Bourgeron, Sven Bölte

**Affiliations:** 1 Center of Neurodevelopmental Disorders Disorders at Karolinska Institutet (KIND), Centre for Psychiatry Research, Department of Women’s and Children’s Health, Karolinska Institutet & Stockholm Health Care Services, Child and Adolescent Psychiatry Stockholm, CAP Research Center, Gävlegatan 22 (Entré B), Floor 8, SE-11330 Stockholm, Sweden; 2 Human Genetics and Cognitive Functions, Institut Pasteur, UMR3571 CNRS, IUF, Université de Paris Cité, Paris, France; 3 Child and Adolescent Psychiatry, Stockholm Health Care Services, Region Stockholm, Stockholm, Sweden; 4 Curtin Autism Research Group, Curtin School of Allied Health, Curtin University, Perth, Western Australia

**Keywords:** Functioning, Screening, Diagnosis, Biopsychosocial, Harmonization

## Abstract

**Purpose:**

A considerable number of screening and diagnostic tools for autism exist, but variability in these measures presents challenges to data harmonization and the comparability and generalizability of findings. At the same time, there is a movement away from autism symptomatology to stances that capture heterogeneity and appreciate diversity. The International Classification of Functioning, Disability and Health (ICF) provides a classification system that can support content harmonization of different screening and diagnostic tools for autism while enabling the translation of diagnostic information into functioning.

**Method:**

Here we linked commonly used screening and diagnostic measures within the field of autism to the ICF to facilitate the unification of data obtained from these measures.

**Results:**

As expected, screening and diagnostic measures primarily focus on body functions and activities and participation domains of the ICF, and much less on environmental factors, reflecting biomedical and adaptive behavior operationalizations of autism derived from diagnostic manuals.

**Conclusion:**

By translating symptomology-based information to the continuous and diagnostically neutral view of functioning, the ICF linking presented here may provide a means to harmonize measures of autism characteristics while enabling diagnostic information to be re-examined through a more neurodiversity-affirmative lens.

**Supplementary Information:**

The online version contains supplementary material available at 10.1007/s10803-023-06204-2.

Autism research is evolving, with bibliometric studies showing a steady increase in the number of publications on autism each year (Rong et al., 2022; Zhao et al., 2022). This rise in research has, among other things, been spurred by increasing diagnosis rates of autism (Zeidan et al., 2022) and international calls for building capacity to support autistic individuals and their families (World Health Organization, 2013). Research and funding in the field covers a broad range of areas, from preclinical to applied research and biomedical to psychosocial and educational services provision (Harris et al., 2021). Although biological and genetic research has appeared to predominate funding (Den Houting & Pellicano, 2019; Harris et al., 2021), there are also upcoming trends in research that more closely align with the priorities of the autistic community such as lifespan issues (Den Houting & Pellicano, 2019).

Regardless of the focus of autism research, adequately characterizing the target group using standardized instruments is essential for the scientific quality, generalizability, and comparability of studies. This issue is arguably of greater importance within the field of autism, given the significant heterogeneity and variability in characteristics and functional outcomes (Howlin & Magiati, 2017; Magiati et al., 2014). Many screening and diagnostic measures exist for autism characteristics, typically centered on evaluating the core diagnostic criteria (“symptoms”) of autism (American Psychiatric Association, 2013; World Health Organization, 2019). Still, they can differ regarding their underlying conceptualization and construction (Charman & Gotham, 2013; Fernandopulle, 2011). Some measures may take a categorical view of autism, while others are based on the notion that autistic traits occur dimensionally across the population (Baron-Cohen et al., 2001; Constantino & Gruber, 2012). Even dimensional autism measures may differ markedly, and the availability and evaluation of measures depending on culture and country vary (Bölte et al., 2011, 2016). The method of administering these measures may also differ, with some administered via clinical interview, observation, or questionnaire, and gathered from a range of informants (Charman & Gotham, 2013).

Variability in measures used to estimate autistic characteristics introduces challenges to data harmonization (i.e., aggregating data from various sources) and comparability of findings. Though samples may be compared or aggregated based on the presence or absence of a diagnosis, interpreting findings that rely primarily on this binary categorization may be less informative, given the differences that may arise between individuals within diagnostic domains (Mandy & Skuse, 2008). Individuals may have varying degrees of functioning within each domain (i.e., social communication and focused interests and behaviors) (Cholemkery et al., 2016), and these differences seem to have different underlying mechanisms at both genetic and neural levels (Bertelsen et al., 2021; Mandy & Skuse, 2008; Warrier et al., 2019; Zabihi et al., 2019). Resultantly, breaking down categorical views of autism and considering heterogeneity is necessary to provide insights into autism (Mandy, 2018; Mandy & Skuse, 2008).

Coinciding with requirements to consider heterogeneity are demands for research to move away from medicalized models of autism to those that recognize and appreciate diversity (Pellicano & den Houting, 2022). The burgeoning neurodiversity movement opposes pathologizing autism and ‘symptomology.’ Instead, it purports that autism is part of natural human variation where disability results from a poor person-environment fit (Bölte, 2022a; den Houting, 2018; Mandy, 2018; Pellicano & den Houting, 2022). Neurodiversity-affirmative research looks beyond symptomology, examines strengths alongside challenges, explores how environments can influence functioning, and tends to align better with the priorities of the autistic community. To be at the forefront of an emerging paradigm shift, researchers must conduct neurodiversity-affirmative research (Pellicano & den Houting, 2022; Sonuga-Barke & Thapar, 2021) and are faced with the challenge of re-imaging how data is understood to facilitate these approaches.

To this end, methods of harmonizing screening and diagnostic measures in a way that enables symptomology to be re-examined through a more neurodiversity-affirmative lens would be beneficial to advancing the field. The bio-psycho-social framework and classification system of the International Classification of Functioning, Disability, and Health (ICF) provide a means to achieve this aim, concerned with functioning, defined as the interaction between an individual, their activities and participation, and their environment (World Health Organization, 2001). According to the ICF, all individuals, regardless of their diagnosis, demonstrate functional strengths and challenges within the context of their environment, which can act to support or disable (World Health Organization, 2001). This conceptualization of functioning aligns readily with a neurodiversity standpoint, integrating social and biomedical understandings of autism (Bölte et al., 2021).

The ICF classification system (World Health Organization, 2001) and its Child and Youth version (ICF-CY; World Health Organization, 2007) contain nearly 1700 codes covering the domains of body functions, body structures, activity and participation, and environmental factors. Codes are organized hierarchically within the four domains, providing up to four levels of increasing detail. To enhance the applicability of the ICF to clinical practice, Core Sets or sets of codes most relevant to capturing the functioning of autistic individuals have also been developed (Bölte et al., 2023; Bölte et al., 2019).

Previous research has used the ICF to explore three diagnostic measures in children, namely the Autism Diagnostic Observation Schedule – Generic (ADOS-G), the Autism Diagnostic Interview- Revised (ADI-R), and the Childhood Autism Rating Scales (CARS) as a means to facilitate documentation of functional information and data integration (Castro et al., 2013). In this current study, we expand and update the previous linking conducted by Castro et al. (2013) and present ICF linking of several commonly used autism screening and diagnostic measures to aid harmonization and to facilitate neurodiversity-affirmative investigation.

## Method

### Selection of Measures

As the purpose of this study was to support harmonization, we reviewed measures applied in large-scale pan-European datasets such as the EU-AIMS Longitudinal European Autism Project (LEAP) (Loth et al., 2017), AIMS-2 Trials Preschool Brain Imaging Project (PIP) (https://www.aims-2-trials.eu/pip/), and Comorbid Analysis of Neurodevelopmental Disorders (CANDY) (https://www.candy-project.eu/). Additional literature was also scoped. Measures were included if they screened, measured, or evaluated autism characteristics.

### ICF Linking

**Fig. 1:**
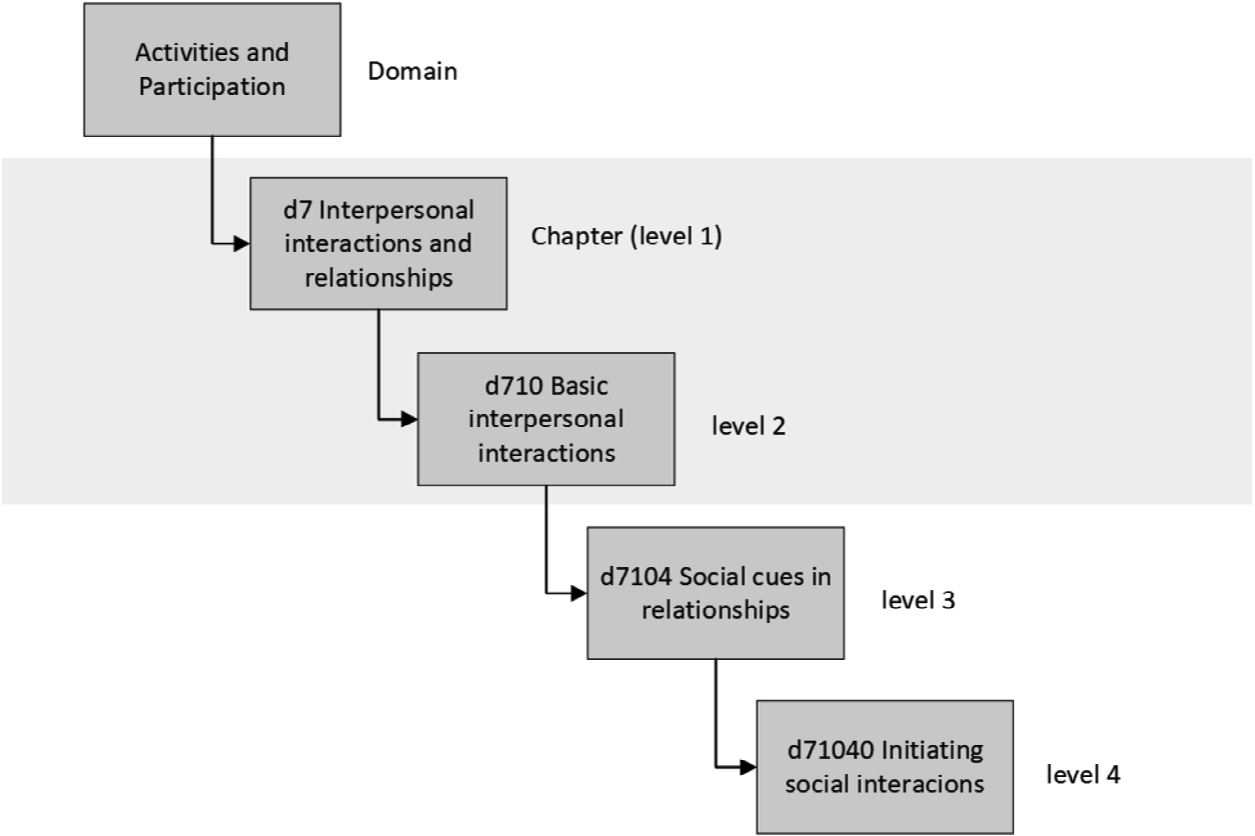
Example of Hierarchical structure of the International Classification of Functioning, Disability and Health (ICF; WHO 2001). The grey box indicates the level of codes presented

Identified measures were linked to the ICF-CY according to linking rules (Cieza et al., 2019). The ICF-CY was used because this version contains additional codes relevant to developing individuals (World Health Organization, 2007) and enables comparisons to the Comprehensive Core Set for autism which is based on the ICF-CY (Bölte et al., 2023; Bölte et al., 2019). First, the purpose of the assessment was identified, and each item and their corresponding response options were extracted. The main and additional concepts were then identified for each item. The main concept referred to “what the item is about” or the most relevant concept, while additional concepts contained other relevant information. Identified concepts were then linked to the most precise ICF-CY category (Fig. 1). Although personal factors (for example, age, gender, coping style) are not officially classified in the ICF, we applied the personal factors classification outlined by Grotkamp et al. (2020) to capture any potential personal factors included in the measures. “Not covered” was used when the item was not covered by the ICF, and “Not definable” was applied when there was insufficient information to decide on the ICF code (Cieza et al., 2019). The extracted data and linking are available in the supplementary file.

One researcher experienced with the ICF and linking methodology conducted the linking. To ensure reliability, a second independent researcher, also experienced with the ICF and linking methodology, completed the linking separately for the Autism Diagnostic Observation Schedule – 2nd Edition (ADOS-2) and the Autism Quotient (AQ). These measures were selected to ensure that different measurement formats (i.e., observation, self-report measure) were subjected to inter-rater reliability. To calculate inter-rater reliability, second-level codes allocated to each item by the two raters were compared and allocated a nominal value based on whether there was agreement. Inter-rater agreement at the second level was 73%, with Cohens Kappa yielding substantial agreement (*k* = 0.73, CIs: *k =* 0.66–0.80). Disagreements were resolved via discussion until consensus was achieved. The frequency distribution of codes at the chapter (first) and second levels of the ICF were calculated and reported, with frequencies presented as a percentage of codes covered within a chapter.

## Results

### Description of Measures

Eleven measures were selected for inclusion, including eight screening and four diagnostic measures. Each measure, including the raters, target population, number of items, and scoring method, are described in Table 1. The ADOS-2, AQ, Social Communication Questionnaire (SCQ), and Social Responsiveness Scale-2 (SRS-2) contain multiple versions designed for different ages (AQ, SRS-2, ADOS-2), expressive language abilities (ADOS-2), or time periods (SCQ). All versions of these measures were linked; however, because only the ADOS-2 and SRS-2 showed a different distribution of codes across each version, only linking for each version of these measures is included in the manuscript; however, linking for each version is available in the supplement. Thus, a total of seventeen measures (including multiple versions of the ADOS-2 and SRS-2) are presented in the summaries.

**Table 1:** Screening and diagnostic measures included in ICF linking

Measure	Type	Rater	Ages	Scoring
***Screening***				
Autism Behavior Checklist (Krug et al., 1980)	Rating	Caregiver or teacher	Children > 3 years	57 items which are assigned a weighted score (1–4) indicating the extent to which the statement describes the individual. Items are organized across five domains: Sensory, relating, body concept, language, social and self-help. Domain scores and a total score are available, with higher scores indicating greater autistic-like traits. Suggested cut-offs: Scores < 47 - indicate typical, 47–53 inconclusive, 54–67 – moderate, 68 ≥ indicative of autism.
Adult Social Behavior Questionnaire (ASBQ) (Horwitz et al., 2016)	Rating	Self-report and person familiar with individual	Adults > 17 years	44 items are rated on a three-point scale indicating the extent to which the statement describes the individual. Items are organized across six domains: Contact, empathy, insight, conventions rigidity, sensory. Domain scores and a total score are available. Higher scores indicate greater autistic-like traits.
Autism Quotient (AQ) (child, adolescent, and adult versions) (Auyeung et al., 2008; Baron-Cohen et al., 2001, 2006)	Rating	Caregiver (child and adolescent), self-report	Child version: 4–11 years Adolescent version: 12–15 years Adult version: > 16 years	50 items are rated on a three-point scale indicating extent of agreement that the statement applies to the individual. Items are organized across six domains: Social skill, attention switching, attention to detail, communication, imagination. Scores range from 0–50. Higher scores indicate greater autistic like traits. Suggested cut-off for autism ≥ 32.
Childhood Social Behaviour Questionnaire (CSBQ) (Hartman et al., 2006)	Rating	Caregiver	Children 4–18 years	49 items rated on a three-point scale indicating the extent to which the statement describes the individual. Items assigned a weighted score (1–4) indicating the extent to which the statement describes the individual. Items are organized across six domains: behavior not optimally tuned to the social situation (not tuned), reduced contact and social interest (social), difficulties in understanding social information (understanding), orientation problems in time, place, or activity (orientation), stereotyped behaviors (stereotyped), and fear of and resistance to change (change). Domain and a total score are available. Higher scores indicate greater autistic-like traits.
Modified Checklist for Autism in Toddlers (M-CHAT) (Robins et al., 2014)	Rating	Caregiver	Children 16–30 months	20 items rated according to a binary yes/no indicating the presence of behavior. Scores range from 0–20. Higher scores indicate higher autistic-like traits. Score of 0–2 indicates low likelihood of autism, 3–7 indicates medium likelihood of autism, 8–20 indicates high likelihood of autism.
Quantitative Checklist for Autism in Toddlers (Q-CHAT) (Allison et al., 2008)	Rating	Caregiver	Children 1–24 months	25 items rated on a four-point scale indicating the presence or frequency of behavior. Scores range from 0-100 with higher scores indicating greater autistic-like traits. The suggested cut-off point of 39 is indicative of autism.
Social Communication Questionnaire (SCQ) Current and Lifetime forms (Rutter, Bailey, et al., 2003)	Rating	Caregiver	Mental age of at least 2 -years	40 items rated on a binary yes/no indicating the presence of behavior. Provides a total score and subscales are available for three domains: Reciprocal social interaction, communication, and restricted, repetitive, and stereotyped patterns of behavior. Total scores range from 0–39, with higher scores indicating greater autistic-like traits. Scores ≥ 15 on the Lifetime form are indicative of autism.
Social Responsiveness Scale second version (SRS-2) (Adult, school aged version, and preschool versions). (Constantino & Gruber, 2012)	Rating	Self, Caregiver, or Teacher	Preschool version: 2.5–4.5 years School age version: 4–18 years Adult version: > 19 years	65 items rated on a four-point scale indicating the extent to which the statement describes the individual. Five treatment subscales (social awareness, social cognition, social communication, social motivation, and restricted interests and repetitive behavior), two DSM-5 compatible subscales and a total score are available. Total possible raw scores range from 0-195 (self-report and parent) and 0-180 (teacher report). Results are reported as T-scores. T score < 59 indicates low likelihood of social difficulties associated with autism, 60–65 indicate mild to moderate social difficulties, 66–75 indicate moderate/some clinically significant social difficulties,≥ 76 indicates substantial/clinically significant social difficulties.
***Diagnostic***				
Autism Diagnostic Interview – Revised (ADI-R) (Rutter, Le Couteur, et al., 2003)	Interview	Clinician administered	Non-verbal mental age of at least 2 -years	93 items rated on a four-point scale indicating the extent to which behavior is present. Provides scores for three domains: Social interaction, communication and restricted and repetitive behaviors. Scores range from 0–31 (social interaction), 0–28 (communication), 0–25 (restrictive and repetitive behavior). Higher scores indicate a higher likelihood of autism.
Autism Diagnostic Observation Schedule 2nd Edition (ADOS-2) (Modules 1–4) (Lord et al., 2012)	Observation	Clinician administered	Module 1: Children > 31 months with no consistent phrase speech Module 2: Children with phrase speech but not fluent speech Module 3: Verbally fluent children and adolescents Module 4: Verbally fluent older adolescents and adults	28–31 items (dependent on version) rated on a four-point scale indicating the extent to which behavior is present. Provides scores for three domains: Social affect, restricted and repetitive behaviors, and social communication. Social affect: 0–27, restricted and repetitive behaviors 0–18, social communication 0–16.
Childhood Autism Rating Scale (CARS) (Schopler et al., 1980)	Observation	Clinician administered	Children > 2 years	15 items rated on a four-point scale indicating the extent to which behavior is present. Provides a total score that ranges from 15–60. Score < 30 is classified as no autistic traits/low likelihood of autism, a score of 30–36indicates mild to moderate traits/likelihood of autism, and a score of ≥ 37 indicates substantial traits/likelihood of autism.
Diagnostic Interview for Social and Communication Disorders (DISCO) (Wing et al., 2002)	Interview	Clinician administered	Children and adults	320 items rated on four-point scale indicating the extent to which behavior is present. Measures following domains: social interaction, communication, repetitive behaviors, and restrictive interests, play and imagination, motor skills and coordination, adaptive functioning, emotional and behavioral regulation, cognitive abilities, sensory sensitives and social relationships.

### Linking Results

**Fig. 2:**
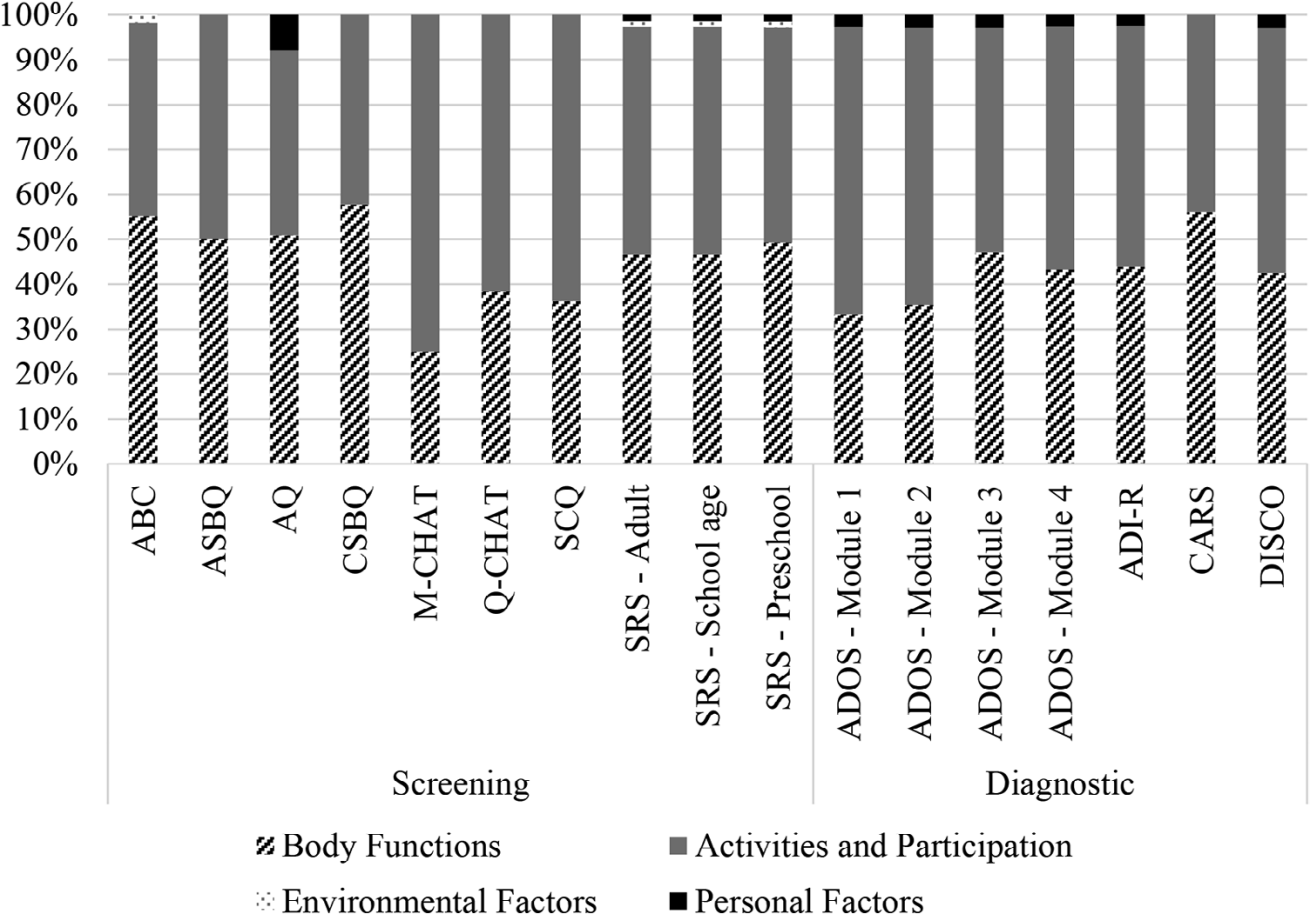
Distribution of ICF codes across domains for each measure *Note. Body structures are not included because no measure was linked to this domain. ABC – Autism Behavior Checklist, ASBQ – Adult Social Behavior Questionnaire, AQ - Autism Quotient, CSBQ - Child Social Behavior Questionnaire, M-CHAT - Modified Checklist for Autism in Toddlers, Q-CHAT - Quantitative Checklist for Autism in Toddlers, SCQ – Social Communication Questionnaire, SRS – Social Responsiveness Scale, ADOS - Autism Diagnostic Observation Schedule-2, M – Module, ADI-R – Autism Diagnostic Interview – Revised, CARS – Childhood Autism Rating Scale, DISCO –Diagnostic Interview for Social and Communication Disorders*

A total of 940 main and additional concepts were extracted from the measures, which were linked to 1150 ICF and 29 personal factor codes. A small number of concepts were coded as Not definable (n = 28) or Not covered (n = 77). The distribution of ICF codes across domains is displayed in Fig. 2, and linking at the chapter level for screening and diagnostic measures are shown in Tables 2 and 3, respectively.

**Table 2:** Linking of screening measures at the chapter level. Percentages represent the distribution of codes across the ICF domains and chapters

	ABC	ASBQ	AQ	CSBQ	M-CHAT	Q-CHAT	SCQ	SRS - Adult	SRS - School	SRS - Preschool
**Total codes applied**	58	56	51	52	20	26	44	73	73	69
**Body Functions (BF)**										
**N codes**	32	28	26	30	5	10	16	34	34	34
**% of total codes**	55%	50%	51%	58%	25%	38%	36%	47%	47%	49%
b1 Mental functions										
N codes	20	24	26	27	4	6	14	31	31	31
% within BF domain	63%	86%	100%	90%	80%	60%	88%	91%	91%	91%
b2 Sensory functions and pain										
N codes	2	0	0	0	0	0	0	0	0	0
% within BF domain	6%	0%	0%	0%	0%	0%	0%	0%	0%	0%
b3 Voice and speech functions										
N codes	1	0	0	0	0	1	0	1	1	1
% within BF domain	3%	0%	0%	0%	0%	10%	0%	3%	3%	3%
b7 Neuromusculoskeletal and movement-related functions										
N codes	9	4	0	3	1	3	2	2	2	2
% within BF domain	28%	14%	0%	10%	20%	30%	13%	6%	6%	6%
**Activities and Participation (AP)**										
**N codes**	25	28	21	22	15	16	28	37	37	33
**% of total codes**	43%	50%	41%	42%	75%	62%	64%	51%	51%	48%
d1 Learning and applying knowledge										
N codes	7	3	6	4	3	4	5	6	6	3
% within AP domain	28%	11%	29%	18%	20%	25%	18%	16%	16%	9%
d2 General tasks and demands										
N codes	1	7	2	4	0	1	0	4	4	4
% within AP domain	4%	25%	10%	18%	0%	6%	0%	11%	11%	12%
d3 Communication										
N codes	7	4	6	6	4	4	9	14	12	10
% within AP domain	28%	14%	29%	27%	27%	25%	32%	38%	32%	30%
d4 Mobility										
N codes	0	0	0	0	1	0	0	0	0	0
% within AP domain	0%	0%	0%	0%	7%	0%	0%	0%	0%	0%
d5 Self-care										
N codes	2	0	0	1	0	0	0	1	1	1
% within AP domain	8%	0%	0%	5%	0%	0%	0%	3%	3%	3%
d7 Interpersonal interactions and relationships										
N codes	7	13	6	7	7	7	10	12	13	13
% within AP domain	28%	46%	29%	32%	47%	44%	36%	32%	35%	39%
d8 Major life areas										
N codes	1	0	1	0	0	0	4	0	1	2
% within AP domain	4%	0%	5%	0%	0%	0%	14%	0%	3%	6%
d9 Community, social and civic life										
N codes	0	1	0	0	0	0	0	0	0	0
% within AP domain	0%	4%	0%	0%	0%	0%	0%	0%	0%	0%
**Environmental Factors (EF)**										
**N codes**	1	0	0	0	0	0	0	1	1	1
**% of total codes**	2%	0%	0%	0%	0%	0%	0%	1%	1%	1%
e2 Natural environment and human-made changes to environment										
N codes	1	0	0	0	0	0	0	0	0	0
% within EF domain	100%	0%	0%	0%	0%	0%	0%	0%	0%	0%
e4 Attitudes										
N codes	0	0	0	0	0	0	0	1	1	1
% within EF domain	0%	0%	0%	0%	0%	0%	0%	100%	100%	100%
**Personal Factors (PF)**										
**N codes**	0	0	4	0	0	0	0	1	1	1
**% of total codes**	0%	0%	8%	0%	0%	0%	0%	1%	1%	1%
i4 Attitudes, action-related skills, and behavior patterns										
N codes	0	0	4	0	0	0	0	1	1	1
% within PF domain	0%	0%	100%	0%	0%	0%	0%	100%	100%	100%

*Note*: Only linked chapters are displayed. ABC – Autism Behavior Checklist, ASBQ – Adult Social Behavior Questionnaire, AQ - Autism Quotient, CSBQ - Child Social Behavior Questionnaire, M-CHAT - Modified Checklist for Autism in Toddlers, Q-CHAT - Quantitative Checklist for Autism in Toddlers, SCQ – Social Communication Questionnaire, SRS – Social Responsiveness Scale

**Table 3:** Linking of diagnostic measures at the chapter level. Percentages represent the distribution of codes across the ICF domains and chapters

	ADOS -M1	ADOS - M2	ADOS - M3	ADOS - M4	ADI-R	CARS	DISCO
**Total codes applied**	36	34	34	37	82	25	268
**Body Functions (BF)**							
**N codes**	12	12	16	16	36	14	114
**% of total codes**	33%	35%	47%	43%	44%	56%	43%
b1 Mental functions							
N codes	9	8	12	12	27	14	83
% within BF domain	75%	67%	75%	75%	75%	100%	73%
b2 Sensory functions and pain							
N codes	0	0	0	0	0	0	1
% within BF domain	0%	0%	0%	0%	0%	0%	1%
b3 Voice and speech functions							
N codes	1	2	2	2	4	0	4
% within BF domain	8%	17%	13%	13%	11%	0%	4%
b4 Functions of the cardiovascular, haematological, immunological and respiratory systems							
N codes	0	0	0	0	1	0	1
% within BF domain	0%	0%	0%	0%	3%	0%	1%
b5 Functions of the cardiovascular, haematological, immunological and respiratory systems							
N codes	0	0	0	0	0	0	2
% within BF domain	0%	0%	0%	0%	0%	0%	2%
b7 Neuromusculoskeletal and movement-related functions							
N codes	2	2	2	2	4	0	23
% within BF domain	17%	17%	13%	13%	11%	0%	20%
**Activities and Participation (AP)**							
**N codes**	23	21	17	20	44	11	146
**% of total codes**	64%	62%	50%	54%	54%	44%	54%
d1 Learning and applying knowledge							
N codes	2	3	2	2	7	4	32
% within AP domain	9%	14%	12%	10%	16%	36%	22%
d2 General tasks and demands							
N codes	1	1	1	2	3	1	9
% within AP domain	4%	5%	6%	10%	7%	9%	6%
d3 Communication							
N codes	9	5	6	8	12	3	26
% within AP domain	39%	24%	35%	40%	27%	27%	18%
d4 Mobility							
N codes	0	0	0	0	2	0	7
% within AP domain	0%	0%	0%	0%	5%	0%	5%
d5 Self-care							
N codes	0	0	0	0	3	1	26
% within AP domain	0%	0%	0%	0%	7%	9%	18%
d6 Domestic life							
N codes	0	0	0	0	0	0	3
% within AP domain	0%	0%	0%	0%	0%	0%	2%
d7 Interpersonal interactions and relationships							
N codes	10	11	8	8	12	1	35
% within AP domain	43%	52%	47%	40%	27%	9%	24%
d8 Major life areas							
N codes	1	1	0	0	5	1	5
% within AP domain	4%	5%	0%	0%	11%	9%	3%
d9 Community, social and civic life							
N codes	0	0	0	0	0	0	3
% within AP domain	0%	0%	0%	0%	0%	0%	2%
**Personal Factors (PF)**							
**N codes**	1	1	1	1	2	0	8
**% of total codes**	3%	3%	3%	3%	2%	0%	3%
i4 Attitudes, action-related skills, and behavior patterns							
N codes	1	1	1	1	2	0	8
% within PF domain	100%	100%	100%	100%	100%	0%	100%

*Note*: Only linked chapters are displayed. ADOS - Autism Diagnostic Observation Schedule-2, M – Module, ADI-R – Autism Diagnostic Interview – Revised, CARS – Childhood Autism Rating Scale, DISCO –Diagnostic Interview for Social and Communication Disorders

#### Body Functions

**Fig. 3:**
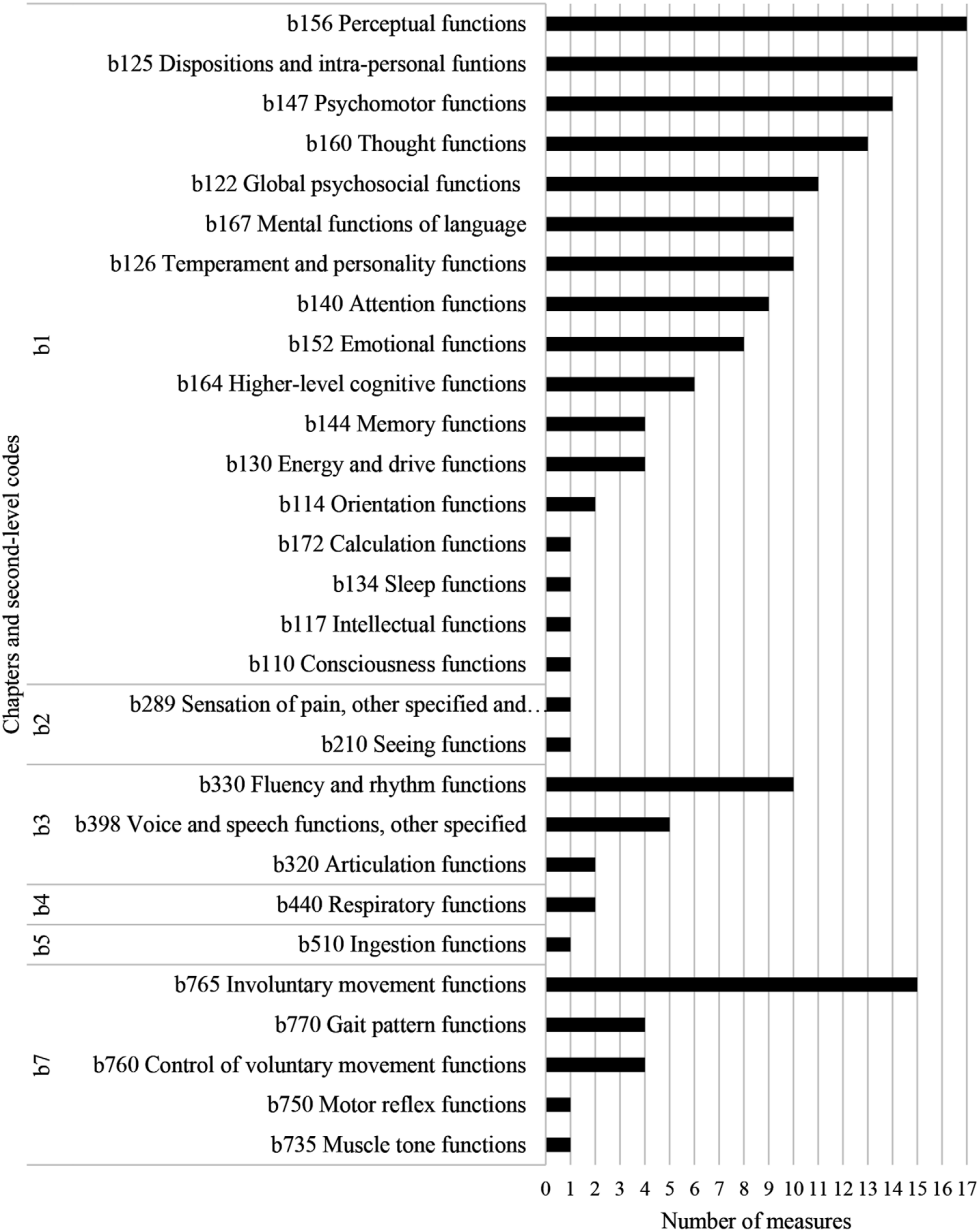
Number of measures covering second level codes within the body functions Chaps. (17 included measures in total, ADOS represents 4 measures and SRS-2 represents 3 measures). Only linked codes are displayed

Figure 3 shows the second-level codes of the body function domain (b) most frequently covered by the measures. All measures were linked to the mental functions chapter (b1). Within this chapter, perceptual functions (b156), describing the perception of sensory information, was the most frequently occurring code. Dispositions and intrapersonal functions was this chapter’s second most frequent code, describing individual dispositions of an individual, including approachability and adaptability. In contrast, temperament and personality functions (b126), describing an individual’s level of extraversion, agreeableness, and openness to experience, were covered by 10 measures. Psychomotor functions (b147), capturing aspects of psychomotor agitation was also frequently linked. Thought functions (b160), most often describing control of thought related to interests and routines, and global psychosocial functions (b122), capturing mental functions required for developing interpersonal skills and forming relationships, were covered by 13 and 11 measures respectively. Mental functions of language (b167), referring to idiosyncratic use of language was linked to 10 measures, nine measures were linked to attention functions (b140), which included sharing and sustaining attention, and eight measures were linked to emotion functions (b152), which included regulation and appropriateness of emotion.

Neuromusculoskeletal and movement-related functions (b7) was the second most frequently occurring body functions chapter. Within this chapter, involuntary movement functions were most commonly covered (15 measures), describing various mannerisms, such as flapping, hand and finger mannerisms, and echoing. The voice and speech functions chapter (b3) was covered by over half of the measures, most often referring to fluency and rhythm functions of the voice (b330).

#### Activities and Participation

**Fig. 4:**
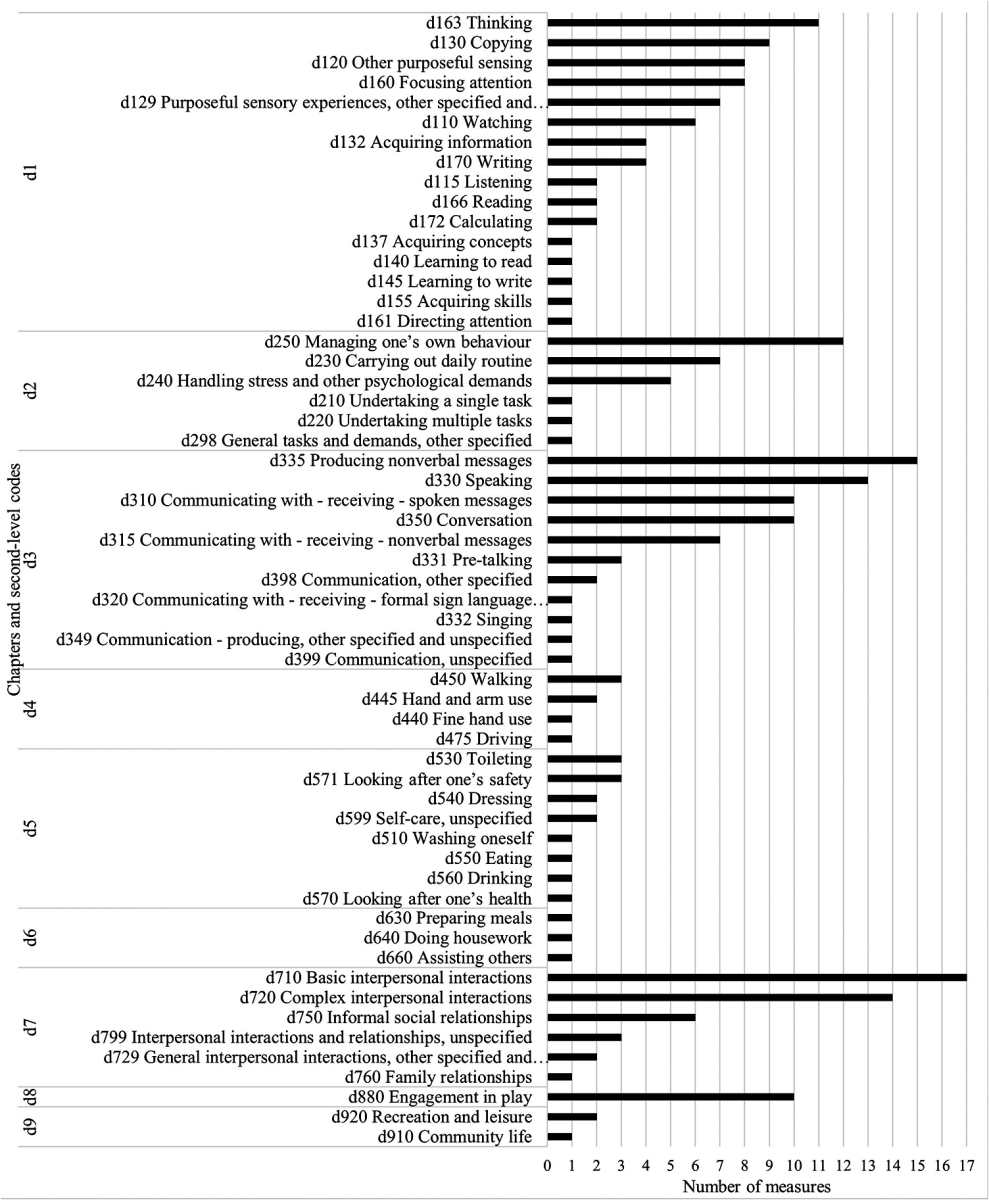
Number of measures covering second level codes within the activity and participant Chaps. (17 included measures in total, ADOS represents 4 measures and SRS-2 represents 3 measures). Only linked codes are displayed

Figure 4 shows the second-level codes most frequently covered by the measures in the activities and participation domain (d). Learning and applying knowledge (d1), communication (d3) and interpersonal interactions and relationships (d7) chapters were linked to all measures. Within the learning and applying knowledge chapter (d1), the most frequently linked code was thinking (d163), which referred to pretending and imagining, while copying (d130) was the second most frequently covered code. Watching (d110) and other sensory experience functions (d120 and d129) codes captured behaviors such as touching, smelling, and visual inspection. Focusing attention was linked to eight measures, referring to the ability to focus attention on individuals or the environment. Within the communication chapter (d3), producing non-verbal messages (d335) and speaking (d330) were linked to most measures. Receiving and understanding spoken (d310) and non-verbal (d315) messages were also commonly linked. Conversation (d350), including beginning, sustaining, and terminating conversations, was linked to 10 measures. Within interpersonal interactions and relationships chapter (d7), basic interpersonal interactions (d710) were linked to all measures. This code captured giving and reacting appropriately to social cues within interactions, responding to physical contact in relationships, differentiating familiar persons and showing and responding to respect, warmth, and appreciation in relationships. Complex interpersonal interactions (d720) were also linked to most measures and included regulating behaviors within relationships, interacting according to social rules and maintaining social space. Six measures were linked to informal social relationships (d750) which refers to developing and maintaining relationships with groups such as peers, families, and friends. General tasks and demands (d2) was covered by most measures. The most linked code was managing one’s own behavior (d250), for example, managing behavior and expression of emotion in response to novelty or demands. The engagement in life areas chapter (d8) was covered by 10 measures, all of which referred to engagement in play (d880). Other chapters such as mobility (d4), self-care (d5), domestic life (d6) and community, social and civic life (d9) were less frequently linked.

#### Environment and Personal Factors

Environment (e) and personal factor (i) domains were rarely covered. Environment codes referred to attitudes of acquaintances, peers, colleagues, neighbors, and community members (e425), which was linked to the three versions of the SRS-2. The ABC was also linked to e240, describing the presence of natural light. Personal factors were linked to 10 measures, most of which were in relation to skills, including methodical skills (i433) and other action-related skills (i448). The DISCO was also linked to eating habits (i450), describing the engagement in “food fads.”

### ICF Core Set Coverage

ICF Core Sets for autism, providing a short list of ICF codes most relevant to functioning in autism exist and have been recently updated (Bölte et al., 2023; Bölte et al., 2019). We thus conduct two additional analyses. First, to assist in validating the content contained in the ICF Core Sets for autism, we examined the percentage of measurement content covered by the ICF Comprehensive Core Set for autism. If the ICF Comprehensive Core Set for autism contains all information relevant to functioning in autism, we would expect much of the measurement content to be contained in the Core Set. We observed a high coverage percentage, with 71–100% of measurement content covered by the ICF Core Sets for autism providing evidence for the validity of the Core Set.

Second, to explore the extent to which the measures capture the breadth of information relevant to functioning in autism, we examined the proportion of the ICF Comprehensive Core Set covered by the measures. As the ICF Comprehensive Core Set for autism is designed to contain all information relevant to functioning in autism, measures that cover a greater degree of the Core Set, capture a greater degree of information relevant to functioning in autism. The proportion of the Core Sets covered by the measures was low, ranging between 8 and 41% of the Core Set. Exploring Core Set coverage across domains showed that measurement coverage of the body function domain ranged between 15 and 65%, 10–55% for the activity and participation domain, and 0–3% for the environment domain (Table 4).

**Table 4:** Comparison of codes contained in measures to the ICF Comprehensive Core Sets for autism based on codes at the second level

	Percentage of measurement content contained in Core Set	Percentage of Core Set covered by measurement (Total)	Percentage of Core Set Body Function domain covered by measure	Percentage of Core Set Activity and Participation domain covered by measure	Percentage of Core Set Environmental factor domain covered by measure
Screening					
ABC	88%	24%	54%	23%	3%
ASBQ	93%	12%	23%	13%	0%
AQ	100%	15%	31%	17%	0%
CSBQ	91%	17%	42%	15%	0%
M-CHAT	92%	9%	15%	12%	0%
Q-CHAT	77%	8%	15%	10%	0%
SCQ	100%	13%	23%	17%	0%
SRS-Adult	79%	21%	42%	23%	3%
SRS-School-aged	85%	23%	46%	25%	3%
SRS-Pre-school	93%	23%	46%	25%	3%
**Diagnostic**					
ADOS - M1	84%	13%	31%	13%	0%
ADOS - M2	89%	14%	31%	15%	0%
ADOS - M3	89%	13%	35%	12%	0%
ADOS - M4	85%	14%	35%	13%	0%
ADI-R	71%	20%	35%	25%	0%
CARS	95%	15%	31%	17%	0%
DISCO	79%	41%	65%	55%	0%

ABC – Autism Behavior Checklist, ASBQ – Adult Social Behavior Questionnaire, AQ - Autism Quotient, CSBQ - Child Social Behavior Questionnaire, M-CHAT - Modified Checklist for Autism in Toddlers, Q-CHAT - Quantitative Checklist for Autism in Toddlers, SCQ – Social Communication Questionnaire, SRS – Social Responsiveness Scale, ADOS - Autism Diagnostic Observation Schedule-2, M – Module, ADI-R – Autism Diagnostic Interview – Revised, CARS – Childhood Autism Rating Scale, DISCO –Diagnostic Interview for Social and Communication Disorders

## Discussion

Unifying different screening and diagnostic measures in autism is necessary for advancing the field, but dimensional views that align with increasing demands for neurodiversity-affirmative research are required. We use the ICF to standardize commonly used autism screening and diagnostic measures in research to facilitate opportunities for the aggregation and comparability of samples while enabling more neurodiversity-affirmative methods.

### Insights for Harmonization and Neurodiversity-Affirmative Approaches

The linking shows areas where items may be readily comparable or harmonized based on functioning rather than symptomatology. Screening and diagnostic measures are most concerned with distinguishing autistic from non-autistic individuals, with a mindset focused on identifying areas of ‘abnormality.’ Conversely, functioning is neutral, lies on a continuum, and can be applied to all individuals, regardless of diagnosis (World Health Organization, 2001). Diagnostic information translated to functioning thus moves away from pathologized views that look at impairment and ‘symptom severity’ to instead look at functioning across domains that may vary from individual to individual. Thus, standardizing autism screening and diagnostic measures using the ICF provides a means to convert data to the more neurodiversity-aligning and neutral view of functioning (Bölte, 2022b), potentially providing avenues for more neurodiversity-affirmative investigation. For example, research could examine clusters based on functioning profiles where one group may have strengths in certain areas of functioning (i.e., perception or attention, commonly observed in autism), and another group may have functional challenges.

The continuous view of functioning also enables more fine-grained investigation that moves beyond diagnostic status, which alone cannot capture the entirety of an individual’s strengths or challenges (Bölte, 2022b) and is insufficient to provide insights into autism’s underlying mechanisms and outcomes (Mandy & Skuse, 2008). Examination instead of functional profiles may assist in decomposing the heterogeneity seen in autism by enabling exploration of how an individual’s functioning across particular domains (for example, attention, memory, environmental support) may be related to specific mechanisms or outcomes, similar to dimensional methods proposed by transdiagnostic approaches (Astle et al., 2022). Beyond providing insights into the nature of autism, this approach also presents important avenues for precision approaches, especially when applied to large data sets to enable the stratification of individuals into more homogenous groups (Loth et al., 2016). Precision approaches based on functional profiles may enable support to be better targeted to an individual’s unique needs and situations.

Harmonization based on the ICF can support data aggregation across projects and studies to facilitate the generation of larger databases. One of the more apparent advantages of aggregating datasets is the increased sample size offered (Adhikari et al., 2021), which can be a key factor limiting analysis based on sub-types or other characteristics in autism research (Lombardo et al., 2019). The ICF has particular utility in harmonization since it is a universally accepted framework that can be applied across multiple contexts, ranging from clinical practice, research, education, and policy (World Health Organization, 2001). The common language provided may also facilitate transdisciplinary approaches, which may be crucial to translating clinical or biomedical research into real-world outcomes, such as between neuroscience and education or examining the interlinks between genetics and clinical outcome (Arnett et al., 2019). The need for more information on functioning beyond diagnosis is also highlighted by recent studies showing that loss-of-function genetic variations in autism and neurodevelopmental condition-associated genes are detected in undiagnosed individuals from the general population (Rolland et al., 2023). Transdisciplinary approaches are also important for sharing knowledge between disciplines to develop new insights that may not be obtainable by one discipline alone. By supporting transdisciplinary research by applying a common language and classification system, data sharing may be facilitated, and new avenues for investigation may be uncovered.

#### Insights for Screening and Diagnostic Measurements in Autism

Our linking also presents insights and future directions on information captured by currently available screening and diagnostic measures. Given the core diagnostic criteria, it is not surprising that there is considerable overlap in the coverage of ICF categories between screening and diagnostic measures, with the linking results largely aligning with diagnostic criteria for autism (American Psychiatric Association, 2013; World Health Organization, 2019). Variability was, however, observed in the areas covered within these domains. For example, all measures were linked to basic social interactions, but fewer were linked to relationship codes that capture entering, forming, and maintaining relationships. Given that a core diagnostic criterion for autism is difficulties in developing, maintaining, and understanding relationships (American Psychiatric Association, 2013; World Health Organization, 2019), it is unexpected that these domains showed limited coverage in the measures. This perhaps reflects a tendency for measures to focus on an individual’s abilities (i.e., eye contact) rather than how any individual may perform within the context of an interaction or relationship. As research has shown that autistic individuals may have differing communication styles than neurotypical individuals and that the success of an exchange may be, at least in part, dependent upon the neurotype of a communication partner (Crompton et al., 2020), exploring an individuals’ functioning within the context of relationships may be necessary to consider.

The fact that measures covered only a limited proportion of the Core Sets for autism indicates that these measures based on diagnostic criteria do not capture all factors important to autistic functioning. Few measures were linked to environment codes, reflective of medicalized approaches that place functional difficulties as resulting from individual challenges. Acknowledging the role of the environment is, however, emphasized by the ICF (World Health Organization, 2001) and neurodiversity paradigm (den Houting, 2018). Exploring the role of the environment may be necessary to capture to understand the true functioning of autistic individuals.

Though not part of diagnostic criteria, some measures examined functions related to mobility and gait patterns. Movement difficulties are commonly reported in autistic populations (Gandotra et al., 2020; Licari et al., 2020), with some suggesting that motor difficulties may be required as a specifier within diagnostic criteria (Licari et al., 2020). Motor functioning may thus be an important consideration, especially when exploring outcomes regarding daily participation. Also not included in diagnostic criteria are the strengths that autism may bring in certain instances, such as superior visual processing and attention (de Schipper et al., 2015). Few measures captured these strengths, and a more explicit focus on abilities alongside challenges may provide a more accurate depiction of an individual’s functioning. Capturing strengths is also a recommendation of more recent diagnostic recommendations (Whitehouse et al., 2018). Differences in the ICF coverage are, in part, reflective of the overall purpose of the measures, for instance, the DISCO was developed to obtain developmental history and functioning more broadly, whereas other measures are more specifically focused on autism symptomology.

It should be noted that the intention of this linking exercise presented is not to question the necessity or utility of screening and diagnostic measures. Diagnostic status remains important in many areas, particularly in funding models, and thus remains necessary to capture. Linking to the ICF however, provides additional and novel ways through which information collected via these measures may be viewed. Examining the range of functions covered within a particular measure may be of interest to researchers and clinicians when selecting measures, where there may be a desire to employ measures that cover a broader range of domains that can inform more decisions around functioning. This information may be helpful to consider alongside psychometric information when selecting screening and measurement tools for research and clinical practice.

#### Limitations

Findings should be interpreted with the following limitations. Measures such as the ADI-R and DISCO are designed to be used in an interview form. Therefore, respondents may discuss other factors pertinent to functioning during the interview. The linking presented here may thus represent the minimum functional information obtainable from these measures. Some nuance between items were likely lost in the linking process, for example, items referring to features such as “sharing enjoyment in interaction,” “giving,” and “eye-contact” were all coded as “basic interpersonal interactions other specified.” Nevertheless, the linking presented still presents a means to retain greater nuance in profiles compared to more traditional harmonization methods. Though the linking process is guided by linking rules, and despite high inter-rater agreement, ICF linking remains subjective and may be influenced by linker knowledge and background. Finally, though linking to the ICF can support more neurodiversity-affirmative views by translating information embedded in symptomology or “disorder” to the diagnostically neutral and continuous view of functioning, linking of existing measures alone will not solely capture all aspects necessary for truly neurodiversity-affirmative research. Linking relies on the content of original measures, and thus, though it is capable of capturing functional strengths and environmental impacts on functioning if it is not covered within the measures, it will be under-represented in the translated data. This can be observed in the linking of measures in the current study, where environmental factors are poorly represented.

Steps must be taken to harmonize the scales quantitatively. Two potential solutions may be implemented to address the quantitative harmonization of autism screening and diagnostic measures based on the ICF. The WHO encourages the use of qualifiers to quantify the severity of a problem ranging from “no problem” to “complete problem” or “mild barrier/facilitator” to “complete barrier/facilitator” in the case of environmental factors (World Health Organization, 2001). This harmonization approach may be suitable for screening and diagnostic measures embedded primarily in areas of difficulties. Where strengths are captured, the use of qualifiers may be less appropriate. Instead, scales could be converted to an 11-point Likert scale ranging from “no difficulty” to “complete strength” or “complete difficulty” (Mahdi et al., 2018). Other harmonization methods, such as applying standardized scores (e.g., z-transformation), may also be appropriate.

## Conclusion

We present the linking of commonly used autism screening and diagnostic measures to the ICF. It is anticipated that this will support data harmonization and data sharing while simultaneously enabling symptomatology to be re-examined through lens of functioning. The more fine-grained and dimensional view of functioning may present avenues for more neurodiversity affirmative investigation.

## Electronic supplementary material

Below is the link to the electronic supplementary material.

Supplementary Material 1
